# Ethyl 2-(1,3-benzodioxol-5-yl)-1-[3-(2-oxopyrrolidin-1-yl)prop­yl]-1*H*-benz­imidazole-5-carboxyl­ate

**DOI:** 10.1107/S1600536812001420

**Published:** 2012-01-21

**Authors:** Yeong Keng Yoon, Mohamed Ashraf Ali, Ang Chee Wei, Safra Izuani Jama Asik, Ibrahim Abdul Razak

**Affiliations:** a Institute for Research in Molecular Medicine, Universiti Sains Malaysia, Minden 11800, Penang, Malaysia; b School of Physics, Universiti Sains Malaysia, 11800 USM, Penang, Malaysia

## Abstract

In the title compound, C_24_H_25_N_3_O_5_, the benzimidazole and benzodioxole ring systems are each approximately planar [maximum deviations = 0.043 (1) and 0.036 (1) Å, respectively]. Their mean planes form a dihedral angle of 42.85 (4)°. The pyrrolidine ring has an envelope conformation with one of the methyl­ene C atoms forming the flap. In the crystal, weak C—H⋯O hydrogen bonds link the mol­ecules into a three-dimensional network. The crystal packing is further stabillized by weak π–π inter­actions between the benzene rings within the benzimidazole ring system [centroid–centroid distance = 3.7955 (7) Å]. A weak C—H⋯π inter­action involving the benzodioxole ring is also present.

## Related literature

For the pharmacological appplications of benzimidazole derivatives, see: Grassmann *et al.* (2002 ▶); Demirayak *et al.* (2002 ▶); Evans *et al.* (1997 ▶). For ring conformation analysis, see: Cremer & Pople (1975 ▶). For the stability of the temperature controller used in the data collection, see: Cosier & Glazer (1986 ▶). For related structures, see: Yoon *et al.* (2012*a* ▶,*b* ▶,*c* ▶)
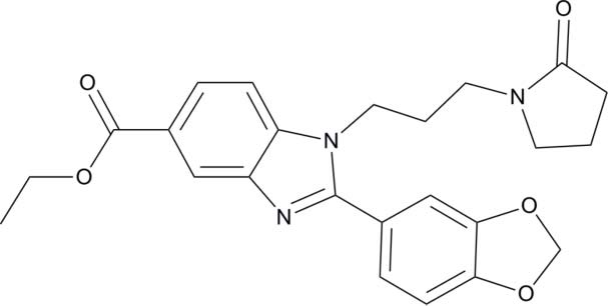


## Experimental

### 

#### Crystal data

C_24_H_25_N_3_O_5_
*M* *_r_* = 435.47
Monoclinic, 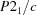

*a* = 11.1692 (2) Å
*b* = 11.5498 (2) Å
*c* = 17.4607 (3) Å
β = 109.210 (1)°
*V* = 2127.05 (6) Å^3^
*Z* = 4
Mo *K*α radiation
μ = 0.10 mm^−1^
*T* = 100 K
0.50 × 0.49 × 0.21 mm

#### Data collection

Bruker SMART APEXII CCD area-detector diffractometer
Absorption correction: multi-scan (*SADABS*; Bruker, 2009 ▶) *T*_min_ = 0.954, *T*_max_ = 0.980
50292 measured reflections
7050 independent reflections
5762 reflections with *I* > 2σ(*I*)
*R*_int_ = 0.032

#### Refinement

*R*[*F* ^2^ > 2σ(*F* ^2^)] = 0.050
*wR*(*F* ^2^) = 0.134
*S* = 1.04
7050 reflections
290 parameters
H-atom parameters constrained
Δρ_max_ = 0.45 e Å^−3^
Δρ_min_ = −0.38 e Å^−3^

### 

Data collection: *APEX2* (Bruker, 2009 ▶); cell refinement: *SAINT* (Bruker, 2009 ▶); data reduction: *SAINT*; program(s) used to solve structure: *SHELXTL* (Sheldrick, 2008 ▶); program(s) used to refine structure: *SHELXTL*; molecular graphics: *SHELXTL*; software used to prepare material for publication: *SHELXTL* and *PLATON* (Spek, 2009 ▶).

## Supplementary Material

Crystal structure: contains datablock(s) global, I. DOI: 10.1107/S1600536812001420/lh5402sup1.cif

Structure factors: contains datablock(s) I. DOI: 10.1107/S1600536812001420/lh5402Isup2.hkl

Supplementary material file. DOI: 10.1107/S1600536812001420/lh5402Isup3.cml

Additional supplementary materials:  crystallographic information; 3D view; checkCIF report

## Figures and Tables

**Table 1:** Hydrogen-bond geometry (Å, °) *Cg* is the centroid of the C8–C13 ring.

*D*—H⋯*A*	*D*—H	H⋯*A*	*D*⋯*A*	*D*—H⋯*A*
C5—H5*A*⋯O5^i^	0.95	2.49	3.4324 (15)	172
C15—H15*B*⋯O3^ii^	0.99	2.52	3.4288 (18)	153
C21—H21*A*⋯O3^iii^	0.99	2.28	3.184 (2)	151
C24—H24*B*⋯O3^iv^	0.99	2.43	3.338 (2)	153
C16—H16*B*⋯*Cg*^ii^	0.98	2.80	3.702 (2)	154

Symmetry codes: (i) 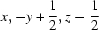; (ii) 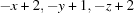; (iii) 
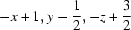
; (iv) 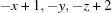.

**Fig. 1.**
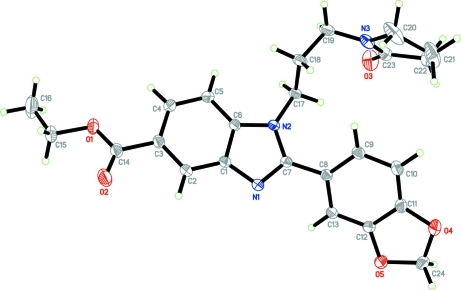
The molecular structure of the title compound, showing 50% probability displacement ellipsoids. Hydrogen atoms are shown as spheres of arbitrary radius.

**Fig. 2.**
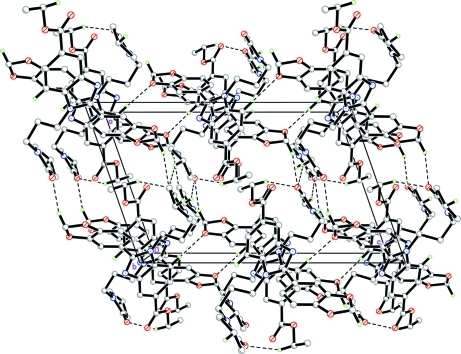
The crystal packing, viewed along the b-axis, showing the molecules are connected into three-dimensional network. Hydrogen bonds are shown as dashed lines.

## supplementary crystallographic information

### Comment

2-substituted benzimidazoles are proven important drug leads. They have therefore generated pharmacological interests (Grassmann *et al.*, 2002; Demirayak *et al.*, 2002; Evans *et al.*, 1997). As part of our ongoing structural studies of benzimidazole derivatives (Yoon *et al.*, 2012*a*,*b*,*c*), we now report the structure of the title compound (I).

In (I), the benzimidazole, (N1–N2/C1–C7) and benzodioxole, (O4–O5/C8–C13/C24) rings are approximately planar with a maximum deviation of 0.043 (1) Å at atom N1 and 0.036 (1) Å at atom O4, respectively. The mean plane through the benzimidazole ring makes a dihedral angle of 42.85 (4) ° with the mean plane through the benzodioxole ring. The pyrrolidine ring adopts an envelope conformation with puckering parameters, Q = 0.2094 (17) Å and φ = 64.7 (4)° and atom C21 at the flap (Cremer & Pople, 1975).

In the crystal (Fig. 2), intermolecular C5—H5A···O5(x,-y+1/2,z-1/2), C15—H15B···O3(2-x,1-y,2-z), C21—H21A···O31(-x,-1/2+y,3/2-z) and C24—H24B···O3(1-x,-y,2-z) interactions link the molecules into a three-dimensional network. In addition, π–π interactions are observed which involve the benzimidazole ring system between the benzene, (C1–C6;centroid *Cg*1) rings with a *Cg*1···*Cg*1(2-x,1-y,2-z) distance of 3.7955 (7) Å. The crystal packing is further stabilized by weak C—H···π interactions (Table 1) involving the benzodioxole rings.

### Experimental

Ethyl 3-amino-4-(3(2-oxopyrrolidin-1yl)propylamino)benzoate (0.84 mmol) and sodium metabisulfite adduct of piperonal (1.68 mmol) were dissolved in DMF. The reaction mixture was reflux at 403K for 2 hrs. After completion, the reaction mixture was diluted in ethyl acetate (20 mL) and washed with water (20 mL). The organic layer was collected, dried over Na_2_SO_4_ and the evaporated in vacuo to yield the product. The product was recrystallised from ethyl acetate.

### Refinement

All the H atoms were positioned geometrically and refined using a riding-model approximation with with C–H = 0.95–0.99 Å. The *U*_iso_ values were constrained to be 1.5*U*_eq_ (methyl-H atom) and 1.2*U*_eq_ (other H atoms). The rotating model group was applied for the methyl group.

### Crystal data

**Table:**

C_24_H_25_N_3_O_5_	*F*(000) = 920
*M**_r_* = 435.47	*D*_x_ = 1.360 Mg m^−^^3^
Monoclinic, *P*2_1_/*c*	Mo *K*α radiation, λ = 0.71073 Å
Hall symbol: -P 2ybc	Cell parameters from 9946 reflections
*a* = 11.1692 (2) Å	θ = 2.5–31.5°
*b* = 11.5498 (2) Å	µ = 0.10 mm^−^^1^
*c* = 17.4607 (3) Å	*T* = 100 K
β = 109.210 (1)°	Block, yellow
*V* = 2127.05 (6) Å^3^	0.50 × 0.49 × 0.21 mm
*Z* = 4	

### Data collection

**Table:**

Bruker SMART APEXII CCD area-detector diffractometer	7050 independent reflections
Radiation source: fine-focus sealed tube	5762 reflections with *I* > 2σ(*I*)
graphite	*R*_int_ = 0.032
φ and ω scans	θ_max_ = 31.5°, θ_min_ = 1.9°
Absorption correction: multi-scan (*SADABS*; Bruker, 2009)	*h* = −13→16
*T*_min_ = 0.954, *T*_max_ = 0.980	*k* = −16→16
50292 measured reflections	*l* = −23→25

### Refinement

**Table:**

Refinement on *F*^2^	Primary atom site location: structure-invariant direct methods
Least-squares matrix: full	Secondary atom site location: difference Fourier map
*R*[*F*^2^ > 2σ(*F*^2^)] = 0.050	Hydrogen site location: inferred from neighbouring sites
*wR*(*F*^2^) = 0.134	H-atom parameters constrained
*S* = 1.04	*w* = 1/[σ^2^(*F*_o_^2^) + (0.0625*P*)^2^ + 0.8818*P*] where *P* = (*F*_o_^2^ + 2*F*_c_^2^)/3
7050 reflections	(Δ/σ)_max_ < 0.001
290 parameters	Δρ_max_ = 0.45 e Å^−^^3^
0 restraints	Δρ_min_ = −0.38 e Å^−^^3^

### Special details

**Table:**

Experimental. The crystal was placed in the cold stream of an Oxford Cryosystems Cobra open-flow nitrogen cryostat (Cosier & Glazer, 1986) operating at 100.0 (1) K.
Geometry. All esds (except the esd in the dihedral angle between two l.s. planes) are estimated using the full covariance matrix. The cell esds are taken into account individually in the estimation of esds in distances, angles and torsion angles; correlations between esds in cell parameters are only used when they are defined by crystal symmetry. An approximate (isotropic) treatment of cell esds is used for estimating esds involving l.s. planes.
Refinement. Refinement of F^2^ against ALL reflections. The weighted R-factor wR and goodness of fit S are based on F^2^, conventional R-factors R are based on F, with F set to zero for negative F^2^. The threshold expression of F^2^ > 2sigma(F^2^) is used only for calculating R-factors(gt) etc. and is not relevant to the choice of reflections for refinement. R-factors based on F^2^ are statistically about twice as large as those based on F, and R- factors based on ALL data will be even larger.

### Fractional atomic coordinates and isotropic or equivalent isotropic displacement parameters (Å^2^)

**Table:**

	*x*	*y*	*z*	*U*_iso_*/*U*_eq_	
O1	1.36916 (8)	0.64900 (8)	1.00135 (6)	0.02630 (19)	
O2	1.46990 (9)	0.52246 (9)	1.09862 (6)	0.0351 (2)	
O3	0.53054 (11)	0.16025 (9)	0.79853 (8)	0.0412 (3)	
O4	0.75646 (11)	−0.15864 (9)	1.14027 (6)	0.0373 (2)	
O5	0.83670 (11)	−0.00736 (10)	1.23064 (6)	0.0413 (3)	
N1	1.08459 (9)	0.25301 (8)	1.08845 (5)	0.01863 (18)	
N2	0.94409 (9)	0.27310 (8)	0.96239 (5)	0.01759 (18)	
N3	0.68842 (9)	0.06821 (9)	0.76740 (6)	0.0242 (2)	
C1	1.12627 (10)	0.33875 (9)	1.04755 (6)	0.0178 (2)	
C2	1.23809 (11)	0.40311 (10)	1.07214 (6)	0.0198 (2)	
H2A	1.3005	0.3910	1.1237	0.024*	
C3	1.25516 (11)	0.48569 (9)	1.01862 (7)	0.0201 (2)	
C4	1.16207 (11)	0.50423 (10)	0.94229 (7)	0.0215 (2)	
H4A	1.1745	0.5640	0.9083	0.026*	
C5	1.05304 (11)	0.43769 (10)	0.91560 (7)	0.0209 (2)	
H5A	0.9915	0.4487	0.8636	0.025*	
C6	1.03832 (10)	0.35349 (9)	0.96927 (6)	0.01764 (19)	
C7	0.97710 (10)	0.21525 (9)	1.03573 (6)	0.01711 (19)	
C8	0.90606 (10)	0.11885 (9)	1.05532 (6)	0.01784 (19)	
C9	0.85386 (11)	0.02908 (10)	1.00098 (7)	0.0210 (2)	
H9A	0.8548	0.0344	0.9469	0.025*	
C10	0.80006 (11)	−0.06882 (10)	1.02441 (7)	0.0235 (2)	
H10A	0.7655	−0.1302	0.9876	0.028*	
C11	0.79959 (11)	−0.07201 (10)	1.10277 (7)	0.0230 (2)	
C12	0.84914 (11)	0.01780 (11)	1.15672 (7)	0.0231 (2)	
C13	0.90412 (11)	0.11341 (10)	1.13575 (7)	0.0207 (2)	
H13A	0.9394	0.1734	1.1737	0.025*	
C14	1.37606 (12)	0.55209 (10)	1.04449 (7)	0.0231 (2)	
C15	1.48387 (12)	0.71957 (11)	1.02515 (8)	0.0285 (3)	
H15A	1.5551	0.6761	1.0169	0.034*	
H15B	1.5076	0.7413	1.0831	0.034*	
C16	1.45570 (18)	0.82402 (18)	0.97368 (14)	0.0613 (6)	
H16A	1.5290	0.8762	0.9902	0.092*	
H16B	1.3817	0.8636	0.9798	0.092*	
H16C	1.4377	0.8017	0.9168	0.092*	
C17	0.83289 (10)	0.25829 (10)	0.88998 (6)	0.0194 (2)	
H17A	0.7936	0.3348	0.8720	0.023*	
H17B	0.7697	0.2093	0.9034	0.023*	
C18	0.86814 (11)	0.20207 (12)	0.82103 (7)	0.0253 (2)	
H18A	0.9210	0.2562	0.8020	0.030*	
H18B	0.9186	0.1313	0.8413	0.030*	
C19	0.74954 (12)	0.17057 (12)	0.75023 (7)	0.0282 (3)	
H19A	0.6892	0.2362	0.7390	0.034*	
H19B	0.7733	0.1570	0.7011	0.034*	
C20	0.72393 (14)	−0.04871 (14)	0.75131 (11)	0.0436 (4)	
H20A	0.8155	−0.0627	0.7791	0.052*	
H20B	0.7041	−0.0617	0.6924	0.052*	
C21	0.64389 (16)	−0.12635 (13)	0.78542 (12)	0.0522 (5)	
H21A	0.6121	−0.1940	0.7497	0.063*	
H21B	0.6939	−0.1544	0.8402	0.063*	
C22	0.53501 (15)	−0.05070 (12)	0.78894 (9)	0.0363 (3)	
H22A	0.4576	−0.0664	0.7423	0.044*	
H22B	0.5162	−0.0642	0.8398	0.044*	
C23	0.58101 (12)	0.07147 (11)	0.78574 (7)	0.0249 (2)	
C24	0.77364 (13)	−0.11782 (12)	1.22069 (8)	0.0297 (3)	
H24A	0.8254	−0.1736	1.2611	0.036*	
H24B	0.6904	−0.1096	1.2288	0.036*	

### Atomic displacement parameters (Å^2^)

**Table:**

	*U*^11^	*U*^22^	*U*^33^	*U*^12^	*U*^13^	*U*^23^
O1	0.0228 (4)	0.0255 (4)	0.0303 (4)	−0.0072 (3)	0.0083 (3)	0.0011 (3)
O2	0.0270 (5)	0.0331 (5)	0.0367 (5)	−0.0086 (4)	−0.0010 (4)	0.0027 (4)
O3	0.0344 (6)	0.0334 (5)	0.0616 (7)	0.0032 (4)	0.0238 (5)	−0.0088 (5)
O4	0.0471 (6)	0.0343 (5)	0.0314 (5)	−0.0208 (5)	0.0142 (4)	−0.0004 (4)
O5	0.0557 (7)	0.0490 (6)	0.0199 (4)	−0.0313 (5)	0.0135 (4)	−0.0020 (4)
N1	0.0190 (4)	0.0193 (4)	0.0163 (4)	−0.0022 (3)	0.0041 (3)	−0.0008 (3)
N2	0.0172 (4)	0.0206 (4)	0.0138 (4)	−0.0022 (3)	0.0035 (3)	−0.0012 (3)
N3	0.0183 (4)	0.0263 (5)	0.0240 (5)	0.0014 (4)	0.0014 (4)	−0.0073 (4)
C1	0.0186 (5)	0.0187 (4)	0.0157 (4)	−0.0011 (4)	0.0051 (4)	−0.0018 (3)
C2	0.0195 (5)	0.0209 (5)	0.0173 (5)	−0.0017 (4)	0.0037 (4)	−0.0025 (4)
C3	0.0204 (5)	0.0197 (5)	0.0208 (5)	−0.0036 (4)	0.0078 (4)	−0.0036 (4)
C4	0.0241 (5)	0.0222 (5)	0.0196 (5)	−0.0022 (4)	0.0092 (4)	0.0010 (4)
C5	0.0211 (5)	0.0241 (5)	0.0168 (5)	−0.0013 (4)	0.0052 (4)	0.0010 (4)
C6	0.0171 (5)	0.0194 (4)	0.0163 (4)	−0.0017 (4)	0.0053 (4)	−0.0020 (4)
C7	0.0180 (5)	0.0182 (4)	0.0149 (4)	−0.0002 (4)	0.0051 (4)	−0.0014 (3)
C8	0.0163 (4)	0.0200 (5)	0.0168 (4)	−0.0012 (4)	0.0048 (4)	−0.0009 (4)
C9	0.0215 (5)	0.0233 (5)	0.0193 (5)	−0.0024 (4)	0.0081 (4)	−0.0041 (4)
C10	0.0232 (5)	0.0222 (5)	0.0259 (5)	−0.0046 (4)	0.0092 (4)	−0.0061 (4)
C11	0.0195 (5)	0.0233 (5)	0.0254 (5)	−0.0043 (4)	0.0062 (4)	0.0012 (4)
C12	0.0224 (5)	0.0288 (6)	0.0168 (5)	−0.0061 (4)	0.0047 (4)	0.0009 (4)
C13	0.0200 (5)	0.0245 (5)	0.0165 (4)	−0.0050 (4)	0.0045 (4)	−0.0019 (4)
C14	0.0242 (5)	0.0221 (5)	0.0242 (5)	−0.0045 (4)	0.0094 (4)	−0.0040 (4)
C15	0.0238 (6)	0.0273 (6)	0.0345 (6)	−0.0083 (5)	0.0097 (5)	−0.0031 (5)
C16	0.0378 (9)	0.0587 (11)	0.0704 (12)	−0.0228 (8)	−0.0052 (8)	0.0337 (10)
C17	0.0162 (5)	0.0259 (5)	0.0143 (4)	−0.0013 (4)	0.0027 (4)	−0.0016 (4)
C18	0.0206 (5)	0.0383 (6)	0.0175 (5)	−0.0066 (5)	0.0069 (4)	−0.0073 (4)
C19	0.0254 (6)	0.0418 (7)	0.0159 (5)	−0.0068 (5)	0.0047 (4)	−0.0046 (5)
C20	0.0256 (6)	0.0396 (8)	0.0529 (9)	0.0102 (6)	−0.0042 (6)	−0.0242 (7)
C21	0.0411 (8)	0.0223 (6)	0.0667 (11)	0.0032 (6)	−0.0181 (8)	−0.0037 (7)
C22	0.0425 (8)	0.0291 (6)	0.0320 (7)	−0.0088 (6)	0.0053 (6)	0.0026 (5)
C23	0.0251 (6)	0.0255 (5)	0.0227 (5)	0.0001 (4)	0.0062 (4)	−0.0014 (4)
C24	0.0274 (6)	0.0341 (6)	0.0250 (6)	−0.0085 (5)	0.0054 (5)	0.0079 (5)

### Geometric parameters (Å, °)

**Table:**

O1—C14	1.3373 (15)	C10—C11	1.3704 (17)
O1—C15	1.4588 (15)	C10—H10A	0.9500
O2—C14	1.2071 (15)	C11—C12	1.3880 (17)
O3—C23	1.2256 (16)	C12—C13	1.3706 (16)
O4—C11	1.3672 (14)	C13—H13A	0.9500
O4—C24	1.4330 (17)	C15—C16	1.475 (2)
O5—C12	1.3738 (14)	C15—H15A	0.9900
O5—C24	1.4399 (16)	C15—H15B	0.9900
N1—C7	1.3235 (14)	C16—H16A	0.9800
N1—C1	1.3878 (14)	C16—H16B	0.9800
N2—C6	1.3788 (14)	C16—H16C	0.9800
N2—C7	1.3825 (14)	C17—C18	1.5290 (16)
N2—C17	1.4618 (14)	C17—H17A	0.9900
N3—C23	1.3402 (16)	C17—H17B	0.9900
N3—C19	1.4450 (17)	C18—C19	1.5289 (17)
N3—C20	1.4606 (17)	C18—H18A	0.9900
C1—C2	1.3943 (15)	C18—H18B	0.9900
C1—C6	1.4067 (15)	C19—H19A	0.9900
C2—C3	1.3916 (16)	C19—H19B	0.9900
C2—H2A	0.9500	C20—C21	1.519 (3)
C3—C4	1.4112 (16)	C20—H20A	0.9900
C3—C14	1.4880 (16)	C20—H20B	0.9900
C4—C5	1.3847 (16)	C21—C22	1.515 (2)
C4—H4A	0.9500	C21—H21A	0.9900
C5—C6	1.3971 (15)	C21—H21B	0.9900
C5—H5A	0.9500	C22—C23	1.5090 (18)
C7—C8	1.4714 (15)	C22—H22A	0.9900
C8—C9	1.3968 (15)	C22—H22B	0.9900
C8—C13	1.4130 (15)	C24—H24A	0.9900
C9—C10	1.4031 (16)	C24—H24B	0.9900
C9—H9A	0.9500		
			
C14—O1—C15	115.14 (10)	O1—C15—H15B	110.3
C11—O4—C24	105.89 (10)	C16—C15—H15B	110.3
C12—O5—C24	105.71 (10)	H15A—C15—H15B	108.6
C7—N1—C1	104.97 (9)	C15—C16—H16A	109.5
C6—N2—C7	106.35 (9)	C15—C16—H16B	109.5
C6—N2—C17	124.13 (9)	H16A—C16—H16B	109.5
C7—N2—C17	129.52 (9)	C15—C16—H16C	109.5
C23—N3—C19	123.21 (11)	H16A—C16—H16C	109.5
C23—N3—C20	113.14 (12)	H16B—C16—H16C	109.5
C19—N3—C20	122.67 (12)	N2—C17—C18	111.41 (9)
N1—C1—C2	129.80 (10)	N2—C17—H17A	109.3
N1—C1—C6	109.95 (9)	C18—C17—H17A	109.3
C2—C1—C6	120.20 (10)	N2—C17—H17B	109.3
C3—C2—C1	117.74 (10)	C18—C17—H17B	109.3
C3—C2—H2A	121.1	H17A—C17—H17B	108.0
C1—C2—H2A	121.1	C19—C18—C17	111.01 (10)
C2—C3—C4	121.13 (10)	C19—C18—H18A	109.4
C2—C3—C14	117.42 (10)	C17—C18—H18A	109.4
C4—C3—C14	121.44 (10)	C19—C18—H18B	109.4
C5—C4—C3	121.78 (10)	C17—C18—H18B	109.4
C5—C4—H4A	119.1	H18A—C18—H18B	108.0
C3—C4—H4A	119.1	N3—C19—C18	111.70 (10)
C4—C5—C6	116.43 (10)	N3—C19—H19A	109.3
C4—C5—H5A	121.8	C18—C19—H19A	109.3
C6—C5—H5A	121.8	N3—C19—H19B	109.3
N2—C6—C5	131.70 (10)	C18—C19—H19B	109.3
N2—C6—C1	105.79 (9)	H19A—C19—H19B	107.9
C5—C6—C1	122.50 (10)	N3—C20—C21	103.78 (13)
N1—C7—N2	112.92 (9)	N3—C20—H20A	111.0
N1—C7—C8	121.52 (9)	C21—C20—H20A	111.0
N2—C7—C8	125.53 (9)	N3—C20—H20B	111.0
C9—C8—C13	120.17 (10)	C21—C20—H20B	111.0
C9—C8—C7	122.70 (10)	H20A—C20—H20B	109.0
C13—C8—C7	116.78 (9)	C22—C21—C20	105.03 (12)
C8—C9—C10	121.55 (10)	C22—C21—H21A	110.7
C8—C9—H9A	119.2	C20—C21—H21A	110.7
C10—C9—H9A	119.2	C22—C21—H21B	110.7
C11—C10—C9	117.10 (10)	C20—C21—H21B	110.7
C11—C10—H10A	121.5	H21A—C21—H21B	108.8
C9—C10—H10A	121.5	C23—C22—C21	104.48 (13)
O4—C11—C10	127.99 (11)	C23—C22—H22A	110.9
O4—C11—C12	110.28 (10)	C21—C22—H22A	110.9
C10—C11—C12	121.72 (11)	C23—C22—H22B	110.9
C13—C12—O5	127.89 (11)	C21—C22—H22B	110.9
C13—C12—C11	122.30 (11)	H22A—C22—H22B	108.9
O5—C12—C11	109.79 (10)	O3—C23—N3	124.65 (12)
C12—C13—C8	117.14 (10)	O3—C23—C22	126.37 (13)
C12—C13—H13A	121.4	N3—C23—C22	108.97 (11)
C8—C13—H13A	121.4	O4—C24—O5	108.19 (10)
O2—C14—O1	123.55 (11)	O4—C24—H24A	110.1
O2—C14—C3	123.99 (11)	O5—C24—H24A	110.1
O1—C14—C3	112.46 (10)	O4—C24—H24B	110.1
O1—C15—C16	106.98 (12)	O5—C24—H24B	110.1
O1—C15—H15A	110.3	H24A—C24—H24B	108.4
C16—C15—H15A	110.3		
			
C7—N1—C1—C2	−175.77 (11)	C24—O5—C12—C13	−179.43 (13)
C7—N1—C1—C6	1.68 (12)	C24—O5—C12—C11	−1.18 (15)
N1—C1—C2—C3	−179.21 (11)	O4—C11—C12—C13	177.00 (12)
C6—C1—C2—C3	3.56 (16)	C10—C11—C12—C13	−1.6 (2)
C1—C2—C3—C4	0.43 (16)	O4—C11—C12—O5	−1.37 (15)
C1—C2—C3—C14	−178.59 (10)	C10—C11—C12—O5	180.00 (12)
C2—C3—C4—C5	−3.24 (18)	O5—C12—C13—C8	179.51 (13)
C14—C3—C4—C5	175.73 (11)	C11—C12—C13—C8	1.46 (18)
C3—C4—C5—C6	1.83 (17)	C9—C8—C13—C12	−0.24 (17)
C7—N2—C6—C5	−177.75 (12)	C7—C8—C13—C12	−173.63 (10)
C17—N2—C6—C5	1.48 (18)	C15—O1—C14—O2	−1.08 (18)
C7—N2—C6—C1	0.87 (12)	C15—O1—C14—C3	178.54 (10)
C17—N2—C6—C1	−179.90 (10)	C2—C3—C14—O2	17.46 (18)
C4—C5—C6—N2	−179.30 (11)	C4—C3—C14—O2	−161.55 (12)
C4—C5—C6—C1	2.27 (17)	C2—C3—C14—O1	−162.16 (10)
N1—C1—C6—N2	−1.60 (12)	C4—C3—C14—O1	18.83 (16)
C2—C1—C6—N2	176.14 (10)	C14—O1—C15—C16	−177.39 (14)
N1—C1—C6—C5	177.18 (10)	C6—N2—C17—C18	72.07 (14)
C2—C1—C6—C5	−5.09 (17)	C7—N2—C17—C18	−108.89 (13)
C1—N1—C7—N2	−1.14 (12)	N2—C17—C18—C19	171.60 (10)
C1—N1—C7—C8	176.98 (10)	C23—N3—C19—C18	105.15 (13)
C6—N2—C7—N1	0.17 (12)	C20—N3—C19—C18	−86.92 (14)
C17—N2—C7—N1	−179.00 (10)	C17—C18—C19—N3	−75.98 (14)
C6—N2—C7—C8	−177.86 (10)	C23—N3—C20—C21	−15.83 (15)
C17—N2—C7—C8	2.97 (18)	C19—N3—C20—C21	175.15 (11)
N1—C7—C8—C9	−135.87 (12)	N3—C20—C21—C22	21.08 (15)
N2—C7—C8—C9	42.00 (16)	C20—C21—C22—C23	−19.27 (16)
N1—C7—C8—C13	37.34 (15)	C19—N3—C23—O3	−8.2 (2)
N2—C7—C8—C13	−144.79 (11)	C20—N3—C23—O3	−177.18 (14)
C13—C8—C9—C10	−0.85 (17)	C19—N3—C23—C22	172.53 (11)
C7—C8—C9—C10	172.14 (11)	C20—N3—C23—C22	3.57 (15)
C8—C9—C10—C11	0.72 (18)	C21—C22—C23—O3	−168.92 (15)
C24—O4—C11—C10	−178.17 (13)	C21—C22—C23—N3	10.31 (15)
C24—O4—C11—C12	3.30 (14)	C11—O4—C24—O5	−3.98 (15)
C9—C10—C11—O4	−177.89 (12)	C12—O5—C24—O4	3.19 (15)
C9—C10—C11—C12	0.49 (18)		

### Hydrogen-bond geometry (Å, °)

**Table:**

Cg is the centroid of the C8–C13 ring.

**Table:**

*D*—H···*A*	*D*—H	H···*A*	*D*···*A*	*D*—H···*A*
C5—H5A···O5^i^	0.95	2.49	3.4324 (15)	172
C15—H15B···O3^ii^	0.99	2.52	3.4288 (18)	153
C21—H21A···O3^iii^	0.99	2.28	3.184 (2)	151
C24—H24B···O3^iv^	0.99	2.43	3.338 (2)	153
C16—H16B···Cg^ii^	0.98	2.80	3.702 (2)	154

Symmetry codes: (i) *x*, −*y*+1/2, *z*−1/2; (ii) −*x*+2, −*y*+1, −*z*+2; (iii) −*x*+1, *y*−1/2, −*z*+3/2; (iv) −*x*+1, −*y*, −*z*+2.
